# Multiple Small Bowel and Colonic Perforations Secondary to Cytomegalovirus Infection in a Newly Diagnosed AIDS Patient

**DOI:** 10.7759/cureus.94935

**Published:** 2025-10-19

**Authors:** Liana Saleh, Omar R Zayed, Francesca D Savona, Mbaga Walusimbi

**Affiliations:** 1 General Surgery, Wright State University, Dayton, USA; 2 General Surgery, Premier Health, Dayton, USA

**Keywords:** cytomegalovirus (cmv), hiv aids, immunocompromised patient, intestinal perforation, opportunistic viral infection, postpartum

## Abstract

Cytomegalovirus (CMV) primarily affects immunocompromised individuals and can rarely involve the small bowel, causing deep ulcerations and microperforations. This is most commonly observed in patients with AIDS and CD4 counts below 50 cells/μL who are not receiving antiretroviral therapy (ART). Due to diagnostic challenges and the risk of severe complications, CMV gastrointestinal (GI) disease is associated with high morbidity and mortality. We report the case of a 38-year-old woman from Ecuador, four months postpartum with an unknown cause of infant demise, who presented with a 15-day history of abdominal pain, diarrhea, vomiting, and hypotension. Two exploratory laparotomies revealed multiple small bowel and colonic microperforations requiring bowel resections. CMV leads to both small bowel and colonic perforations primarily through a combination of direct viral cytopathic effects and ischemic injury secondary to vasculitis. Infectious evaluation revealed a new diagnosis of AIDS, and final surgical pathology was positive for CMV. The patient initially recovered from surgery and was started on appropriate systemic therapy, but ultimately left the hospital against medical advice and was noncompliant with ART. She was readmitted three days later with disseminated intravascular coagulation, GI bleeding, multi-organ failure, and disseminated toxoplasmosis. The patient ultimately passed away approximately 45 days after initial presentation. This case underscores the complexities of managing CMV GI disease. Early recognition of immunity status is crucial to broadening the differential to include opportunistic infections. Timely diagnosis and initiation of therapies with good patient compliance could significantly impact outcomes.

## Introduction

Cytomegalovirus (CMV), or human herpes 5 (HH5), is a virus that commonly causes asymptomatic infection in immunocompetent individuals. Globally, CMV seroprevalence among the general population has been estimated to be around 50%, increasing with age and exposure risk [1]. A large majority of these infections in healthy hosts are asymptomatic, and the remainder typically present with a mononucleosis-like syndrome. However, in immunocompromised patients, CMV can lead to devastating effects, including severe end-organ disease in almost any organ system. The most common manifestations in those with severe immunosuppression include retinitis, pneumonitis, encephalitis, and gastrointestinal (GI) tract disease [2].

Although GI involvement by CMV is a relatively rare complication, it has been well-documented in patients with AIDS and severe immunosuppression [3]. The GI tract may be affected anywhere from the esophagus to the rectum, with the colon being the most frequently involved site, followed by the duodenum, stomach, esophagus, and, very rarely, the small intestine [4]. Patients with advanced HIV/AIDS, especially those with CD4 counts <50 cells/μL and who are not on effective antiretroviral therapy (ART), are at the highest risk for severe CMV GI disease. Clinical presentations vary depending on the site of involvement. For example, CMV colitis often presents with abdominal pain, diarrhea, fever, and hematochezia and is one of the leading causes of lower GI bleeds in AIDS patients. In the upper GI tract, CMV can cause ulcerations leading to odynophagia or epigastric pain [5]. On the other hand, small intestinal CMV disease typically manifests as nonspecific abdominal pain and chronic diarrhea [6]. Even among patients with profound immunosuppression, CMV enteritis leading to full-thickness small bowel perforation has been described only in a few isolated case reports, mostly from the pre-ART era [7-9].

We report a case of CMV enteritis causing multiple small bowel perforations in a young postpartum woman with undiagnosed HIV/AIDS. This case is unique in that it highlights an unusual presentation of advanced AIDS and CMV infection. Additionally, it underscores several important challenges in diagnosis and management, including delayed pathology results, patient noncompliance, and language barriers. We discuss how this case contributes to the existing knowledge and emphasize key lessons for clinical practice.

## Case presentation

A 38-year-old Hispanic female, who had recently immigrated from Ecuador, presented with a 15-day history of worsening abdominal pain, vomiting, profuse diarrhea, and two episodes of hematochezia. She was approximately four months postpartum and had recently lost her newborn to a reported hepatitis-related illness. She had no significant past medical history, did not receive regular medical care, and denied alcohol or illicit drug use. Interpreter services were used throughout her care due to a language barrier.

On initial evaluation, she was febrile (38.5℃), hypotensive (80/50 mmHg) despite fluids, and required vasopressor support. Physical examination revealed diffuse abdominal tenderness with rebound and guarding. Labs revealed leukopenia, normocytic anemia, hyponatremia, hypokalemia, and an elevated serum lactate consistent with shock. A summary of initial laboratory findings is provided in Table 1.

**Table 1:** Key laboratory findings at initial presentation

Laboratory test	Result	Reference range
White blood cell count	2.1 x 10^9^/L	4.0-11.0 x 10^9^/L
Hemoglobin	9.5 g/dL	12-16 g/dL
Sodium	128 mmol/L	135-145 mmol/L
Potassium	2.9 mmol/L	3.5-5.0 mmol/L
Serum lactate	4.1 mmol/L	0.5-2.0 mmol/L

**Figure 1:**
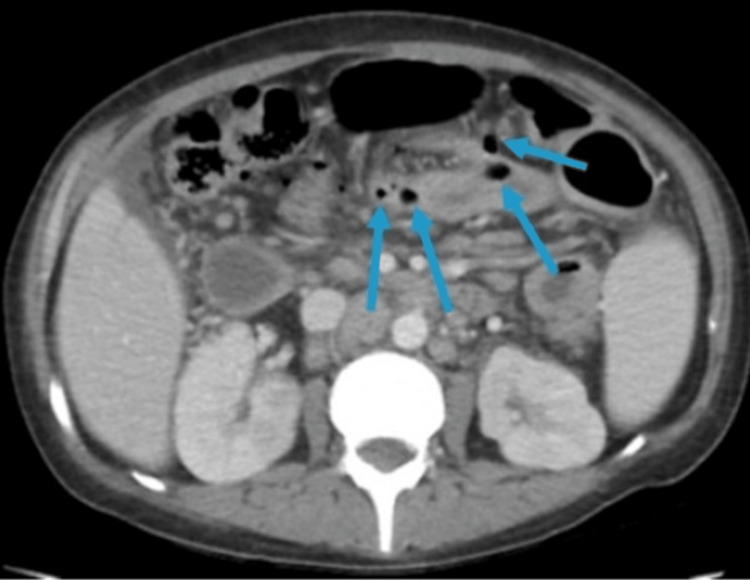
Axial contrast-enhanced CT scan showing severe small bowel enteritis with microperforations (indicated by arrows)

**Figure 2:**
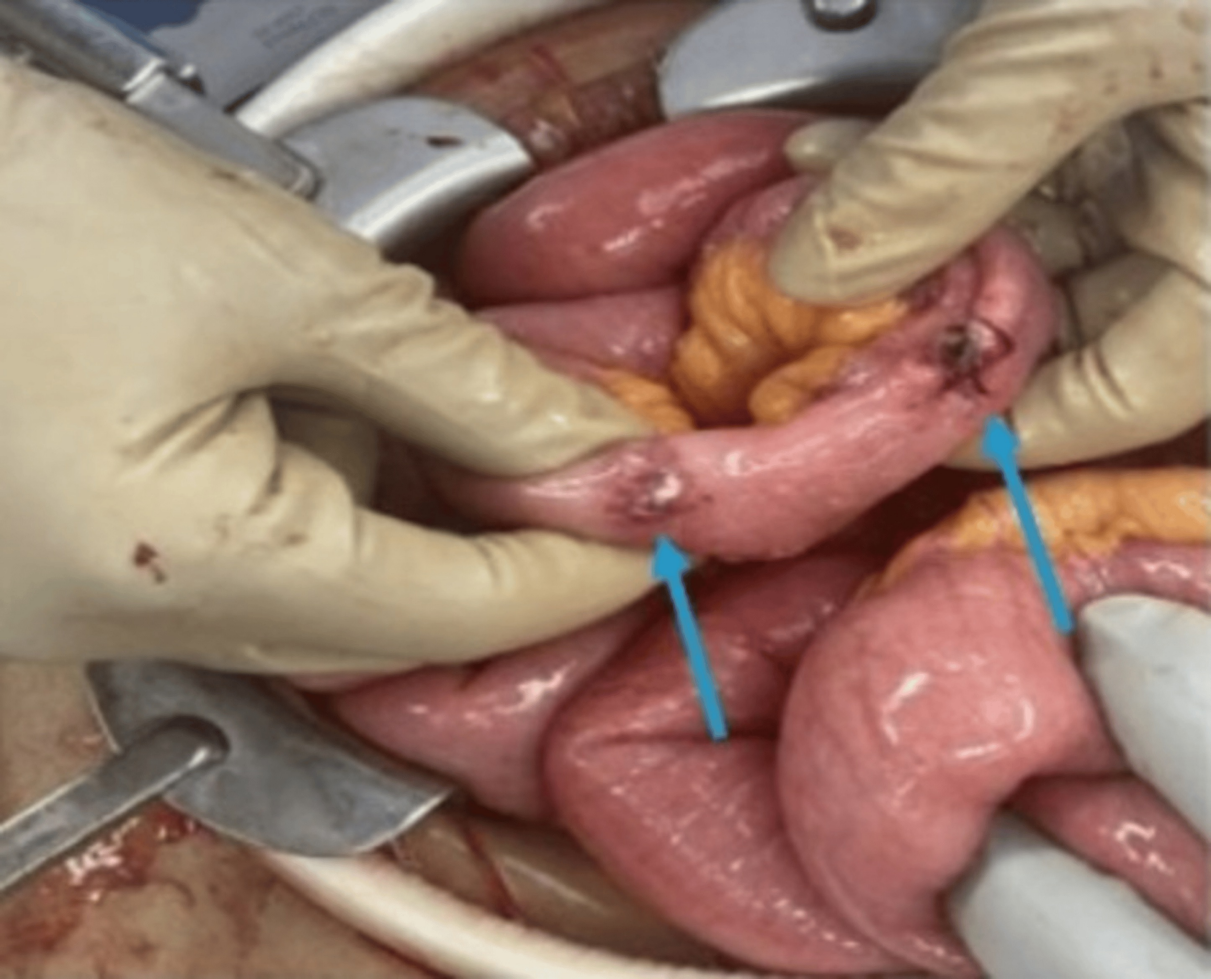
Intraoperative image from the first exploratory laparotomy showing perforations on the small bowel (indicated by arrows)

In the ED, a contrast-enhanced CT of the abdomen and pelvis demonstrated severe small bowel enteritis with lymphadenopathy and microperforations (see Figure 1). She underwent emergent exploratory laparotomy, which revealed several small bowel perforations near the terminal ileum and cecum with fibrinous exudate and adhesions (Figure 2). Approximately 80 cm of small bowel and a portion of the cecum were resected, and the abdomen was temporarily left open due to bowel friability.

In the OR, diagnostic laparoscopy converted to exploratory laparotomy revealed multiple small bowel perforations near the terminal ileum and cecum with significant adhesions and fibrinous exudate (see Figure 2). Postoperatively, the patient required vasopressor support.

The patient underwent re-exploration on postoperative days 2 and 3, requiring further resection and ileocolic anastomosis with definitive closure for additional small bowel and right colonic perforations. Postoperatively, the patient was weaned off pressors and extubated.

In the following days, the patient struggled with confusion, persistent fever spikes, worsening pancytopenia, and continued hypotension. Infectious workup revealed a new diagnosis of AIDS with a viral load of 1,070,000 copies/mL and a CD4 count of 13 cells/μL. Serum and tissue CMV polymerase chain reaction (PCR) were positive, confirming CMV enterocolitis. She was initiated on highly active antiretroviral therapy (HAART), intravenous ganciclovir, broad-spectrum antibiotics, and CMV viral loads were trended. Despite treatment, the patient continued to struggle with hypotension and recurrent fever spikes. Additionally, the patient started to exhibit worsening emotional distress and depressive symptoms and became increasingly noncompliant with medications. Eventually, the patient left the hospital against medical advice.

Three days after leaving, the patient was readmitted with worsening abdominal pain and generalized weakness. She was hypotensive after 2 L of fluid resuscitation and required vasopressor support, consistent with septic shock (lactate 4.2 mmol/L). Labs revealed profound coagulopathy with prothrombin time (PT) > 120 seconds, international normalized ratio (INR) > 16.8, activated partial thromboplastin time (aPTT) > 200 seconds, undetectable fibrinogen, and elevated D-dimer, consistent with disseminated intravascular coagulation (DIC). Hepatic dysfunction (elevated direct bilirubin, aspartate aminotransferase (AST), and alkaline phosphatase) and pancytopenia indicated evolving multi-organ failure. Arterial blood gas demonstrated a pH of 7.42 with low CO_2_ and bicarbonate, consistent with compensated metabolic encephalopathy. The patient was immediately put on broad-spectrum antibiotics.

Over the next few days, the patient remained in critical condition with ongoing hemodynamic instability. The family ultimately transitioned the patient to comfort care, and she passed away. The patient’s initial admission was 17 days long, and the subsequent admission was nine days long.

## Discussion

CMV enteritis is an uncommon yet devastating opportunistic infection seen primarily in severely immunocompromised patients. Since the advent of effective ART, CMV GI disease is rarely encountered as a complication of AIDS, with a decrease in incidence from 40 cases per 1000 person-years before HAART to 4 cases per 1000 person-years [10-13]. However, it still occurs in patients with advanced HIV who are untreated or fail to respond to therapy. Our patient demonstrated the classic risk factors for severe CMV disease, including a CD4 count below 50 and no prior ART coverage. This case highlights the risks of diagnostic delays, patient noncompliance, and social barriers in managing complex infections.

The pathogenesis of CMV GI disease arises from several mechanisms involving direct viral injury, immune-mediated inflammation, and vasculitis. CMV primarily targets intestinal epithelial cells, inducing apoptosis and disruption of tight junctions, which increases transepithelial permeability and promotes bacterial translocation, ultimately weakening the gut wall’s integrity [14,15]. The subsequent inflammatory response driven by CMV-infected monocytes and macrophages further damages the mucosa and submucosa [14-16]. Vasculitis is another key factor, as CMV can infect endothelial cells, leading to microvascular damage and ischemia. This ischemic process can progress to bowel wall necrosis, significantly increasing the risk of perforation [17-19]. Recent transcriptomic and functional analyses further show that CMV profoundly alters monocyte effector functions, downregulating phagocytosis and antigen presentation while inducing a pro-inflammatory cytokine phenotype that amplifies tissue damage and microvascular inflammation [20]. In an immunocompromised patient, such as in this case, an impaired immune response permits extensive viral replication and tissue damage, thereby increasing the risk of perforation [21,22]. The combination of these factors results in a fragile intestinal wall prone to multiple mucosal ulcers, as observed in our patient.

As demonstrated by our patient, the diagnosis of CMV enteritis can be challenging and often delayed. Endoscopic biopsy with histopathology remains the gold standard for diagnosis, which typically reveals the characteristic CMV “inclusion bodies” [19]. Current guidelines recommend specifically immunohistochemical staining or in situ hybridization [23]. The additional modalities that are frequently discussed include PCR and serology testing. Although tissue PCR can be supportive, blood PCR is only sensitive for systemic infection, but not sensitive or specific for GI disease [24,25]. Additionally, patients with CMV enteritis can have negative blood PCR, and viremia does not confirm tissue invasion [26]. Similarly, serology testing in this context is not useful, as it only indicates prior exposure or recent infection but not active tissue-invasive disease [19]. Our patient did have a positive serum CMV PCR with a viral load that only indicated low-level viremia.

The management of CMV enteritis involves antiviral therapy and surgical intervention when necessary. Intravenous ganciclovir is the first-line treatment for CMV disease in immunocompromised patients [23]. In our case, due to high clinical suspicion and positive serum CMV PCR, the care team appropriately started ganciclovir before the release of final pathology results. This may have helped to control the infection to some extent during her first admission. There is evidence that early treatment with antivirals can induce remission and healing of ulcers in CMV colitis [27,28]. However, in our case, there were full-thickness perforations with acute peritonitis requiring surgical intervention. The risks of surgery in these patients are significant, as patients with AIDS and CMV colitis who undergo emergent surgery have high morbidity and mortality [19]. Common postoperative complications include anastomotic leaks, recurrent perforations, and continued sepsis [19]. Our patient did survive the immediate postoperative period and even improved transiently, which could be attributed to successful source control and antiviral therapy. However, our patient eventually discontinued her treatment and left against medical advice. When she was readmitted five days later, she was found to have CMV relapse, suspected disseminated *Toxoplasma*, and other opportunistic infections, which ultimately led to her death. It remains unclear how her clinical course may have differed had she remained hospitalized and continued ART therapy. Sustained treatment and close monitoring may have altered the patient’s outcome.

Additionally, several psychosocial factors contributed to the complexity of treating this patient. Her recent immigration status, limited knowledge of her medical history, and the language barrier posed challenges to establishing rapport, delivering education, and ensuring adherence to treatment. These factors were intensified by the profound emotional trauma of having recently lost her newborn. The degree of suffering and isolation she must have experienced while navigating a new healthcare system in a foreign country was undoubtedly immense and likely played a major role in her leaving against medical advice. This case highlights the importance of multidisciplinary models that include case managers, social workers, and community health workers who can help bridge communication gaps and connect patients to culturally appropriate education.

Prior reports of CMV-related small bowel perforations in AIDS patients describe isolated ileal perforations in profoundly immunosuppressed individuals, typically before or early in the ART era [9,21,22]. In contrast, our patient developed multiple small bowel and colonic perforations within a modern treatment context, highlighting that advanced disease and social barriers can still lead to devastating outcomes despite current diagnostic and therapeutic advances. Contributing to high mortality is the fact that these patients often have multiple simultaneous opportunistic infections, and their organ reserves are poor. Therefore, early HIV diagnosis and treatment are paramount. In patients who do develop CMV GI disease, aggressive antiviral therapy and supportive care are necessary. Notably, there is literature to suggest that surgical resection of affected bowel segments prior to frank perforation may improve outcomes.

## Conclusions

We report a rare and fatal case of CMV enteritis presenting with multiple small bowel perforations in a young woman with previously undiagnosed AIDS. This case highlights three key lessons for clinical practice. First, clinicians must maintain a high index of suspicion for CMV GI disease in immunosuppressed patients presenting with severe abdominal symptoms. Second, early initiation of empiric antiviral therapy can be life-saving, especially when confirmatory pathology is delayed. Third, rapid diagnostic tools such as CMV PCR and immunohistochemistry should be utilized when available to expedite diagnosis. Lastly, this case highlights the profound impact of psychosocial factors such as language barriers, immigration status, trauma, and mental health on treatment adherence and outcomes. In the future, strengthening early HIV screening programs, ensuring timely access to ART, and integrating social support services into HIV care may help prevent such catastrophic presentations in the future.
